# Supporting Hispanic/Latinx Nurses During the COVID-19 Pandemic: Brief Report

**DOI:** 10.1177/1540415320933724

**Published:** 2020-06-24

**Authors:** Norma G. Cuellar, Cresta Archuletta

**Affiliations:** NAHN Executive Director; NAHN Executive Director

We celebrate the culture, caring and spirit of Hispanic nurses who are the leading voice of health in our communities.

As the impact of the COVID-19 pandemic continues to increase across the United States, the needs of our members are a priority for the National Association of Hispanic Nurses (NAHN). Nurses in our organization have voiced a variety of concerns about the challenges they have and are facing. Initially, concerns were related to the safety of our nurses: lack of PPE (personal protective equipment) and the fears associated with contracting SARS-CoV-2, the virus that causes COVID-19 disease. In addition, fears related to transmitting the disease to family members, from the work setting, were also a priority. Other concerns included the negative impact on the mental health of our NAHN members related to the stress and coping with COVID-19, which included working long hours and extended days. In addition, watching so many people who are sick and dying has been and continues to be detrimental to the emotional health of our members. Moreover, situations of incivility and lateral violence in hospitals have been reported.

NAHN is the nation’s leading professional society for Hispanic/Latinx nurses with 49 chapters across the United States. NAHN is devoted to promoting safe, quality health care delivery to Hispanic/Latinx communities and individuals. NAHN addresses health care challenges facing these communities, in this case, our own Latinx health care providers. In response to the increasing concerns voiced by our members, NAHN conducted a series of informal discussions to determine the needs of our members in an effort to initiate interventions to support them. The purpose of this brief report is to convey the initiatives and activities of NAHN that aim to support our members during the COVID-19 pandemic.

## Administrative Activities

To keep the organization running smoothly, NAHN operations and events moved from in-person to online. We determined it was a priority to find ways to support our members that might be in crisis and to mobilize our members to serve and help people in need. From early March to mid-May 2020, the NAHN developed various resources and strategies in response to the COVID-19 pandemic. These resources were refined using feedback from a combination of formal and informal member surveys. NAHN had the additional challenge of working to evaluate, create, and distribute COVID-19 information in Spanish for nurses and the general Spanish-speaking public in the United States. In order to properly create and deploy tools to help our members, we addressed various priorities in the area of Administration and Organization, including the following:

- Development and launch of a bilingual (English/Spanish) COVID-19 resource page on the national website
- Vetting and implementation of secure and reliable virtual meeting platforms
- An extension of the member account expiration grace period from 30 days to 60 days to help mitigate any financial impact to our members
- Policy and Advocacy Activity (see Box 1)
- Management of bilingual national media relations response to crisis
- Research, evaluation, and monitoring related to determining viability of planned in-person conference

**Box 1.** Policy and Advocacy Activity of NAHN During COVID-19 Pandemic.

Drafted NAHN-led COVID-19 Statement Signed on to Nursing Community Coalition (NCC)-led Senate LHHS-ED Written Testimony for the Record. This included (FY) 2021 funding requests for Title VIII and NINR. Signed on to NCC COVID-19 Request Letter to Congress Signed on to Health Equity & Accountability Act of 2020 Support Letter Signed on to Letter to Congress regarding Demographic Data on COVID-19 Patients Signed on to Letter of Concern to HHS/OMH Regarding Unusual Grant Award on COVID-19 Signed on to American Association of Nurse Anesthetists (AANA) led letter to President Trump to address urgent needs for mental health support and care for frontline COVID-19 nurses Signed on to NCC led letter to support full practice authority for CRNAs at the VA

## Mental Health Resources

To specifically address the mental health needs of our members, NAHN began hosting a weekly member support session as well as a monthly self-care series. To provide an intimate and inspirational space for members, NAHN launched a weekly informal virtual chat called, *Wellness Wednesdays: Cafecito Con NAHN*. All members were invited to this password-protected safe space to connect with other NAHN members and share stories, feedback, and even light-hearted conversation. There is no formal presenter or agenda. NAHN staff moderate the call when needed and follow-up on any ideas or requests put forth by meeting participants.

To address the need for a more structured and curriculum-based mental well-being resource, NAHN launched the first in a series of Self-Care Sunday sessions scheduled to take place monthly or bimonthly. NAHN plans to continue the series through the end of the year. At the chapter level, many chapters also initiated a virtual meeting to provide support for their local members.

## Social Media

The pandemic has also required a pivot in NAHN’s digital and social media strategies. New communication strategies include the following:

- Ongoing planning and content creation for a social media campaign focused on support for members during the pandemic
- Planning and execution of two twitter chats (how to support nurses and health equity in the face of COVID-19)
- Planning and promoting a NAHN Engagement Committee campaign called #NAHNSTRONG to feature stories of NAHN members leading in health care

## National Support From Other Organizations

A variety of national organizations have provided assistance to nurses to help during this time. Some of these are include the following.

- *Nurses House* is a nurse-managed, nonprofit organization dedicated to helping nurses in need. They have partnered with the American Nurses Foundation to help nurses nationwide affected by COVID-19. Through July 31, 2020, Nurses House will be accepting application for a grant that will help all nurses (RN and LPN) who are unable to work due to COVID-19 infection, caring for family member, or under quarantine: https://donate.nurseshouse.org/campaigns/14512-help-for-nurses-nationwide-affected-by-covid-19
- The Johnson & Johnson coronavirus website has collated a variety of resources and partnerships: https://www.jnj.com/coronavirus
- Johnson & Johnson Center for Health Worker Innovation, the entity with which we support nurses, midwives, and community health workers around the globe: https://cloud.inform.jnj.com/subscription?qs=75e41ac5e6c608667478eeab4685050591dec9a0e1fcdc138d8c439546b88916d4c5f379a1cab6ed
- Our *First Responders First* collaboration with Harvard and Thrive Global now has brand new resilience resources available at www.thriveglobal.com
- *Headspace*, the app for guided meditation, relaxation, and help with sleep, is now available for all U.S. nurses: https://offers.sheerid.com/headspace/medical
- *Brave of Heart Fund*, funded by the New York Life and Cigna, was established to provide monetary grants to the loved ones of frontline healthcare workers and volunteers who lose their lives because of COVID-19. The grants will provide immediate financial support for common needs such as funeral costs, medical care, counseling, food, education, mortgage payments and living expenses: https://www.braveofheartfund.com/

In conclusion, NAHN has been working diligently to serve our members and our Latinx communities. We are so proud of all our nurses who are working during this pandemic. There is no area of nursing that has not been affected by the pandemic. The summary of the events in this report are just the “tip of the iceberg” and does not represent all the grassroots activities that our members have participated in. It would take an entire journal to report all of the activities.

Special thanks to all NAHN Board of Directors who have worked diligently during this time to make decisions for the organization. We are reminded at this difficult time of the work of Cesar Chavez and Delores Huerta and how they changed the world of Latinx laborers. While the enemy is not against a human industry, it is against an enemy that is worse: still not known, a killer, a mystery. As we come together, believe “Si, se puede,” yes, we can stand together and support each other, and yes, we will get through these times stronger and better for it.

Norma G. Cuellar, PhD, RN, FAAN, NAHNNAHN Immediate Past PresidentCresta Archuletta, BANAHN Executive Director

